# Assessing the level of perceived social support among community-dwelling stroke survivors using the Multidimensional Scale of Perceived Social Support

**DOI:** 10.1038/s41598-022-23840-3

**Published:** 2022-11-11

**Authors:** Shamay S. M. Ng, Tai Wa Liu, Lily Y. W. Ho, Nga Huen Chan, Thomson W. L. Wong, Joshua Tsoh

**Affiliations:** 1 Department of Rehabilitation Sciences, The Hong Kong Polytechnic University, Hung Hom, Hong Kong, Special Administrative Region of China; 2 School of Nursing and Health Studies, Hong Kong Metropolitan University, Ho Man Tin, Hong Kong, Special Administrative Region of China; 3 School of Nursing, The Hong Kong Polytechnic University, Hung Hom, Hong Kong, Special Administrative Region of China; 4 Department of Psychiatry, Prince of Wales Hospital and Shatin Hospital, Shatin, Hong Kong, Special Administrative Region of China

**Keywords:** Health care, Neurology

## Abstract

Social support has an important role in stroke rehabilitation. The Multidimensional Scale of Perceived Social Support (MSPSS) is an instrument examining the adequacy of perceived social support. However, the psychometric properties of the Chinese version of MSPSS (MSPSS-C) have not been examined in Chinese people with stroke. This study aimed at investigating the psychometric properties of the MSPSS-C, identifying the correlations between MSPSS-C scores and health-related measures of these people; and examining the differences in the levels of perceived social support between people with and without stroke in Hong Kong using a cohort of 57 community-dwelling people with stroke and 50 age-matched healthy controls. We found that the MSPSS-C subscales demonstrated excellent internal consistency, and a ceiling effect was observed for the family subscale of the MSPSS-C. The total MSPSS-C score had significant weak to moderate correlations with the scores of the concerned variables of interests. Exploratory factor analysis revealed a two-factor structure for the MSPSS-C. People with stroke had lower levels of perceived social support from friends and their significant other than those without stroke. The MSPSS-C is a valid tool for assessing perceived social support among chronic stroke survivors with moderate to very severe motor impairment.

## Introduction

Social support refers to any support, either received or perceived, accessible to an individual through social ties to other individuals, groups, and the larger community^1^. Perceived social support refers to the perceived availability and adequacy of support received from others^2^. Received social support focuses on the quality and quantity of support received, such as the size of an individual’s social network and the frequency of contact with members of their social network^2^. Social support can be categorised into four constructs—emotional, instrumental, informational, and appraisal support—which can be acquired as received and perceived social support^2,3^. The perception and reception of social support should be assessed independently because perceived support is a measure of how an individual appraises his/her situation, while received support is a reflection of the quantity of support he/she receives^4^.

The level of perceived social support among people with stroke plays an important role in their rehabilitation. Lima et al.^5^ interviewed 108 people with stroke, 74.1% of whom reported medium to low levels of perceived social support. A study demonstrated that increased levels of physical disability were associated with reduced levels of perceived social support in people with stroke (*r* = 0.26, *p* < 0.01)^6^. In addition, higher levels of perceived social support have been demonstrated to improve the functional status of people with stroke^7^ and reduce the fall risk of community-dwelling older adults^8^. Adequate perceived social support can also improve the social participation^9^, and in turn, may improve the functional mobility, motor function, post-stroke depression symptoms^10^, and health-related quality of life of people with stroke^1^. As the level of perceived social support greatly affects the rehabilitation outcome, quality of recovery, and physical and psychosocial well-being of stroke survivors^11,12^, clinicians need a reliable and valid instrument that can measure the levels of perceived social support in these individuals at baseline and help to monitor their progress during a targeted intervention.

Several instruments have been developed to assess perceived social support. For example, the Social Support Questionnaire quantifies perceived social support based on the number of supportive persons listed by the respondents for each item^13^; however, this questionnaire does not identify the sources of support. The Inventory of Social Support Behaviors assesses the frequency at which respondents receive various forms of assistance^14^; however, this inventory does not reflect the subjective perception of social support. The Multidimensional Scale of Perceived Social Support (MSPSS) is a 12-item instrument that examines the adequacy of social support perceived by an individual^15^. The MSPSS was originally developed for adolescents but has also been used for patients with Parkinson’s disease^16^, heart failure^16^, and chronic obstructive pulmonary disease^17^. The main advantage of the MSPSS is that it explicitly operationalises the sources of perceived social support, including family, friends, and one’s significant other, thus allowing clinically relevant information to be obtained. The MSPSS has been translated into different language, such as Swedish^18^, Korean^19^, and French^20^. Generally, the MSPSS across different translations showed a good internal consistency with Cronbach’s α of at least 0.70^21^.

Understanding and enhancing the levels of perceived social support in people with stroke are important for their rehabilitation. However, there is a lack of a comprehensive and validated instrument for this purpose in Chinese people with stroke. Although the MSPSS has been translated into Chinese using adolescents^22^ and may be used as an outcome measure to assess the level of perceived social support following stroke, its psychometric properties have not been examined in Chinese people with stroke, whose cultural and social constructs, such as willingness of seeking social support, are different from those of people in Western and other Asian countries^23^. Moreover, social support has an important role in health status and we expected that a valid clinical measure should be able to reveal to what extent it associates with the various health domains under the International Classification of Functioning, Disability and Health (ICF) framework. Thus, the objectives of this study were to (i) investigate the psychometric properties of the Chinese version of the MSPSS (MSPSS-C), including its internal consistency and structural validity, in community-dwelling Chinese people with stroke; (ii) identify the correlations between MSPSS-C scores and health-related measures, including motor function, psychosocial well-being, and health-related quality of life; and (iii) examine the differences in the levels of perceived social support between people with and without stroke in Hong Kong.

## Methods

### Participants

Fifty-seven people with stroke and 50 people without stroke were recruited from local self-help groups in Hong Kong by convenience sampling. The inclusion criteria for people with stroke were as follows: (1) aged 45 years or above; (2) diagnosed with stroke by magnetic resonance imaging or computed tomography at least 1 year before the start of this study; (3) moderate to very severe motor impairment, with the total Fugl-Meyer Assessment (FMA) score for motor function ≤ 79^24^; (4) an Abbreviated Mental Test score of 7 or above^25^; (5) no comorbid disease that would affect the performance of daily activities; and (6) ability to communicate in Chinese (Cantonese). People with stroke were excluded if they had any unstable medical condition that could hinder proper assessment. The inclusion and exclusion criteria for people without stroke were the same as those for people with stroke, except that people without stroke should have no history of stroke and no motor impairment. The recruited subjects were invited to a university-based neurorehabilitation laboratory where the study was conducted. After informed consent obtained, all the participants were asked to complete a sociodemographic sheet and then all the outcome measures. The stroke participants were administered the MSPSS-C again after a 7-day interval.

### Sample size calculation

To compare the levels of perceived social support between people with and without stroke, we assumed an effect size of 0.50 to avoid over- or under-estimation. According to our calculation using GPower 3.1, for an effect size of 0.50, a power of 0.80, and a significance level of 0.05, a sample size of 51 people with stroke and 51 people without stroke would be required in this study. For factor analysis, the minimal number of participants would be three times the number of items in the instrument^26^. Thus, for the 12-item MSPSS-C, a minimum sample size of 36 (3 × 12) would be required. To increase the robustness, 57 people with stroke and 50 people without stroke were recruited.

### Outcome measures

The International Classification of Functioning, Disability and Health (ICF) has described that the body function and structure, activities and participation, and environmental factors were interacted with each other in disability^27^. Support and relationship was one of the environmental factors in ICF components^27^. To examine the relationship between perceived social support with body function, activity and participation, outcome measures for motor function, psychosocial well-being and health-related quality of life were included in the present study.

### Outcome measures for social support

#### The Chinese version of the Multidimensional Scale of Perceived Social Support (MSPSS-C)

The MSPSS-C contains 12 items to assess the levels of perceived social support among respondents. Each item in the MSPSS-C is rated on a scale ranging from 1 (very strongly disagree) to 7 (very strongly agree), with the total score ranging from 12 to 84^22^. Higher scores indicate higher levels of perceived social support. The items can be divided into three groups indicating the source of support: family, friends, and significant other. In MSPSS-C, the terms ‘special person’ were replaced by ‘a best friend’. The MSPSS-C has demonstrated good internal consistency (Cronbach’s α = 0.89) in adolescents^22^.

#### The Chinese version of the brief 2-way social support scale (Brief 2-Way SSS-C)

The Brief 2-Way SSS-C is a 12-item scale that evaluates the reciprocity of emotional and instrumental social support^28^. It appraises four principal dimensions: giving emotional support, giving instrumental support, receiving emotional support, and receiving instrumental support. Each item (e.g., “There is someone in my life I can get emotional support form”)is rated on a 6-point Likert scale ranging from 0 (not at all) to 5 (always)^28^. The total score ranges from 0 to 60, with higher scores indicating higher levels of giving or receiving social support. The subscales of the Brief 2-Way SSS-C have demonstrated good internal consistency (Cronbach’s α = 0.74–0.88) and good test–retest reliability (intraclass correlation coefficient [ICC] = 0.76–0.81) in our previous study using people with chronic stroke^28^.

### Outcome measures for motor function

#### Fugl-Meyer assessment (FMA)

The FMA was used to assess the motor function, range of motion, and reflex actions of both upper and lower extremities in our participants^29^. It consists of 33 items and 17 items for the assessment of upper and lower extremities, respectively. All items are rated on a 3-point ordinal scale where 0 represents ‘cannot perform’, 1 represents ‘can perform partially’, and 2 represents ‘can perform fully’. The maximum total score is 100, with higher scores indicating better motor control. The FMA has demonstrated excellent test–retest reliability (ICC = 0.96) and inter-rater reliability (ICC = 0.99) in a previous study using people with hemiplegia^30^.

#### Timed up and go (TUG) test

The TUG test was used to assess the level of functional mobility in our participants^31^. It requires the subjects to stand up, walk 3 metres, turn, walk back, and finally sit down. The time taken to complete the task was recorded. A shorter time taken to complete the test indicates better functional performance. The TUG test has shown excellent test–retest reliability (ICC = 0.95) in our previous study using people with chronic stroke^32^.

#### Berg balance scale (BBS)

The BBS was used to measure the ability of our participants to maintain balance during functional activity^33^. It consists of 14 items rated on a 5-point ordinal scale ranging from 0 to 4. The total score ranges from 0 to 56, with higher scores indicating better ability to maintain balance. A score less than 45 indicates a high risk of fall^34^. The BBS has demonstrated excellent inter-rater reliability (ICC = 0.98) and intra-rater reliability (ICC = 0.99) in a previous study using people with stroke^33^.

### Outcome measures for psychosocial well-being

#### The Chinese version of the geriatric depression scale (GDS-15-C)

The GDS-15-C was used to detect the presence of depression in our participants^35^. It consists of 15 questions and requires the respondents to answer ‘Yes’ or ‘No’ based on how they felt over the past week (e.g., “Are you basically satisfied with your life?”). The total score ranges from 0 to 15, with scores above 8 indicating the presence of depression^36^. The GDS-15-C has demonstrated good internal consistency (Cronbach’s α = 0.80) in a previous study using people with stroke^37^.

#### The Chinese version of the community integration measure (CIM-C)

The CIM-C consists of 10 item, such as “I feel like part of this community, like I belong here” and “I know the rules in this community and I can fit in with them”, to evaluate the community participation of an individual^38^. Each statement is rated on a 5-point Likert scale from 1 (always disagree) to 5 (always agree). The total score ranges from 10 to 50, with higher scores indicating better community integration. The CIM-C has demonstrated good internal consistency (Cronbach’s α = 0.84) and test–retest reliability (ICC = 0.84) in our previous study using people with stroke^38^.

### Outcome measure for health-related quality of life

#### The Chinese version of the 12-item short form health survey (SF-12-C)

The 12-item Short Form Health Survey is a 12-item questionnaire to assess the health-related quality of life (e.g., “In general, would you say your health is”) of respondents through two summary scores: physical component summary (PCS) and mental component summary (MCS). Both PCS and MCS scores range from 0 to 100, with higher scores indicating better physical and mental health-related quality of life, respectively. This questionnaire was translated into Chinese (Cantonese) (the SF-12-C) for use in this study. The Chinese version showed good internal consistency (Cronbach’s α = 0.87) in people with stroke. In addition, correlation analyses (Spearman’s correlation coefficient > 0.4) and comparisons of PCS and MCS scores among stroke populations that differed in age and mobility status (*p* < 0.05) demonstrated good convergent and discriminant validity of the SF-12-C.

### Data analysis

The quantitative data were analysed using SPSS version 26.0 (IBM, Armonk, NY, USA). The statistical significance level was set at *p* < 0.05. Descriptive statistics were used to summarise the demographic characteristics of the participants. The normality of the data was checked using the Shapiro–Wilk test. The independent t-test and Mann–Whitney U test were used to compare parametric and nonparametric data, respectively, between participants with and without stroke. Ceiling and floor effects were examined by calculating the percentage of participants who had the highest or lowest scores. More than 15% of the participants attaining the highest or lowest scores would be indicative of ceiling and floor effects, respectively. Correlations of MSPSS-C scores with Brief 2-Way SSS-C, FMA, TUG, BBS, GDS-15-C, CIM-C, and SF-12-C scores were examined using Pearson’s correlation coefficient or Spearman’s correlation coefficient (r_s_) if the data were normally or non-normally distributed, respectively. Correlation strength was defined as weak (r = 0.10–0.39), moderate (r = 0.40–0.69), strong (r = 0.70–0.89), and very strong (r ≥ 0.90)^39^.

The structural validity of the MSPSS-C was examined by exploratory factor analysis. Principal component analysis and scree plot were used to determine the optimal number of factor extraction. Data were extracted using principal axis factoring with varimax rotation to enhance data interpretability.

### Ethical considerations

This study followed the guidelines of the Declaration of Helsinki. Ethical approval was granted by the Human Subjects Ethics Sub-Committee of The Hong Kong Polytechnic University. All participants signed a declaration of written informed consent after the objectives and procedures of the study had been explained to them.

## Results

### Participant characteristics

Fifty-seven people with stroke and fifty people without stroke were recruited in this study. Their demographic characteristics are shown in Table 1.

**Table 1:** Demographics characteristics of the participants.

Characteristics	People with stroke (n = 57)	People without stroke (n = 50)	*p*
Age (Mean ± SD)	63.82 ± 6.36	61.58 ± 7.56	0.098
**Gender, number (%)**			
Male	37 (64.91%)	14 (28.00%)	< 0.001
Female	20 (35.09%)	36 (72.00%)	
Number of times of stroke (Mean ± SD)	1.23 ± 0.78	–	
Years since stroke (Mean ± SD)	7.04 ± 4.44	–	
**Marital status, number (%)**			
Single	8 (14.04%)	7 (14.00%)	0.482
Married	44 (77.19%)	42 (84.00%)	
Widow/widower	4 (7.02%)	1 (2.00%)	
Divorced/separated	1 (1.75%)	0 (0.00%)	
**Employment status, number (%)**			
Employed	1 (1.75%)	13 (26.00%)	< 0.001
Unemployed/retired	56 (98.25%)	37 (74.00%)	
**Mobility status, number (%)**			
Walks unaided	7 (12.28%)	50 (100.00%)	< 0.001
Walks with aid	50 (87.72%)	0 (0.00%)	
**Living arrangement, number (%)**			
Alone	7 (12.28%)	5 (10.00%)	0.709
With family members	50 (87.72%)	45 (90.00%)	

*SD* standard deviation.

### Internal consistency

The internal consistency (Cronbach’s α) values of the family, friends, and significant other subscales of the MSPSS-C were 0.96, 0.95, and 0.94, respectively (Table 2). We found that the deletion of item 1, 2, 5, or 10 increased the Cronbach’s α value of the significant other subscale by 0.01.

**Table 2:** Internal consistency of MSPSS-C in people with stroke.

	Corrected item-total correlation	Alpha if item deleted
**Family: 0.96**		
3. My family really tries to help me	0.66	0.95
4. I get the emotional help and support I need from my family	0.67	0.95
8. I can talk about my problems with my family	0.70	0.95
11. My family is willing to help me make decisions	0.69	0.95
**Friends: 0.95**		
6. My friends really try to help me	0.86	0.95
7. I can count on my friends when things go wrong	0.78	0.95
9. I have friends with whom I can share my joys and sorrows	0.81	0.95
12. I can talk about my problems with my friends	0.84	0.95
**Significant other: 0.94**		
1. There is a special person who is around when I am in need	0.78	0.95
2. There is a special person with whom I can share my joys and sorrows	0.82	0.95
5. I have a special person who is a real source of comfort to me	0.83	0.95
10. There is a special person in my life who cares about my feelings	0.84	0.95

### Ceiling and floor effects

No ceiling or floor effect was found for the total score or scores of the friends and significant other subscales of the MSPSS-C. However, a ceiling effect, but not a floor effect, was observed for the score of the family subscale, as 13 of the 57 subjects (22.8%) had the highest score.

### Correlations between the MSPSS-C and variables of interest

The Shapiro–Wilk test showed that the data were non-normally distributed. Therefore, correlations between the MSPSS-C and variables of interest were examined using Spearman’s correlation coefficient. The total MSPSS-C score had significant weak to moderate correlations with the scores of all of the Brief 2-Way SSS-C subscales (receiving emotional support, r = 0.500, *p* < 0.001; giving emotional support, r = 0.372, *p* = 0.004; receiving instrumental support, r = 0.374, *p* = 0.004; giving instrumental support, r = 0.404, *p* = 0.004), significant weak correlations with the BBS score (r = 0.406, *p* = 0.002) and the GDS-15-C score (r = − 0.289, *p* = 0.029), and a significant moderate correlation with the CIM-C score (r = 0.369, *p* = 0.005) (Table 3). For the SF-12-C, only the family subscale of the MSPSS-C had significant weak correlations with the MCS score (r = 0.278, *p* = 0.04). The total MSPSS-C and subscale scores had no significant correlations with the FMA score or the TUG completion time.

**Table 3:** Correlations between MSPSS-C and other outcome measures.

Variables	Family		Friends		Significant other		Total score	
	r_s_	*p*	r_s_	*p*	r_s_	*p*	r_s_	*p*
**The Chinese version of the brief 2-way social support scale**								
Receiving emotional	0.501**	< 0.001	0.412**	0.001	0.420**	0.001	0.500**	< 0.001
Giving emotional	0.268*	0.044	0.333*	0.011	0.351**	0.007	0.372**	0.004
Receiving instrumental	0.419**	0.001	0.369**	0.005	0.322*	0.014	0.374**	0.004
Giving instrumental	0.266*	0.045	0.409**	0.002	0.430**	0.001	0.404**	0.002
Fugl-Meyer assessment	0.071	0.606	0.132	0.334	0.107	0.433	0.149	0.274
Timed up-and-go test	0.252	0.061	0.087	0.525	0.012	0.930	0.098	0.474
Berg balance scale	0.314*	0.017	0.331*	0.012	0.336*	0.011	0.406**	0.002
The Chinese version of the 15-item geriatric depression scale	− 0.261*	0.050	− 0.279*	0.036	− 0.287*	0.031	− 0.289*	0.029
The Chinese version of the community integration measure	0.379**	0.004	0.331*	0.012	0.270*	0.042	0.369**	0.005
**The Chinese version of the 12-item short form health survey**								
Physical component	− 0.126	0.357	0.015	0.913	0.019	0.887	− 0.038	0.782
Mental component	0.278*	0.038	0.168	0.215	0.203	0.133	0.233	0.084

*p* < 0.05*, *p* < 0.01**.

### Structural validity

The principal component analysis and scree plot suggested a two-factor structure for the MSPSS-C (factor 1: ‘friends’ and factor 2: ‘family’), with the eigenvalue of each factor exceeding 1. The Kaiser–Meyer–Olkin value was 0.84, indicating sampling adequacy^40^. Bartlett’s test of sphericity was significant (*X*^2^(66) = 862.43, *p* < 0.001), suggesting a satisfactory factor analysis^40^. After varimax rotation, the loadings of all 12 items in the two-factor structure were greater than the recommended minimum value of 0.40^41^. Factor 1 (eigenvalue = 7.96) had loadings ranging from 0.82 to 0.92 and accounted for 66.34% of the total variance. Factor 2 (eigenvalue = 2.07) had loadings ranging from 0.88 to 0.92 and accounted for 17.28% of the total variance (Table 4).

**Table 4:** Rotated factor matrix of MSPSS-C based on the principal component analysis with varimax rotation.

Item	Factor	
	1	2
6. My friends really try to help me	0.92	
2. There is a special person with whom I can share my joys and sorrows	0.89	
5. I have a special person who is a real source of comfort to me	0.88	
12. I can talk about my problems with my friends	0.88	
9. I have friends with whom I can share my joys and sorrows	0.85	
10. There is a special person in my life who cares about my feelings	0.84	
7. I can count on my friends when things go wrong	0.83	
1. There is a special person who is around when I am in need	0.82	
3. My family really tries to help me		0.92
4. I get the emotional help and support I need from my family		0.91
8. I can talk about my problems with my family		0.91
11. My family is willing to help me make decisions		0.88
Eigenvalues	7.96	2.07
Variance explained (%)	66.34	17.28

### Level of perceived social support among community-dwelling people with stroke in Hong Kong

Table 5 shows the medians and interquartile ranges of the MSPSS-C subscale scores. Compared with the participants without stroke, those with stroke scored significantly lower on the subscales of friends (*p* = 0.046) and significant other (*p* = 0.022) and on the total MSPSS-C scale (*p* = 0.024) (Table 5).

**Table 5:** Comparisons of MSPSS-C subscales and total score between the people with stroke and without stroke.

Subscale	Median (interquartile range)		Mann–Whitney U	Z	*P*
	People with stroke (n = 57)	People without stroke (n = 50)			
Family	21.00 (12.00)	24.00 (10.00)	1166.50	− 1.630	0.103
Friends	18.00 (11.00)	22.00 (9.00)	1106.50	− 1.995	0.046*
Significant other	19.00 (12.00)	22.00 (11.00)	1058.00	− 2.298	0.022*
Total score	55.00 (32.00)	67.00 (26.00)	1063.00	− 2.262	0.024*

*p* < 0.05*.

Further, among the participants with stroke, those without depressive symptoms (GDS score < 8) had significantly higher scores on the subscales of family (*p* = 0.022) and significant other (*p* = 0.032) and on the total MSPSS-C scale (*p* = 0.030) than those with depressive symptoms (GDS score ≥ 8) (Table 6). Moreover, among the participants with stroke, those with low fall risks (BBS score ≥ 45) had significantly higher family subscale scores (*p* = 0.003) and total scale scores (*p* = 0.014) than those with high fall risks (BBS score < 45) (Table 7).

**Table 6:** Comparisons of MSPSS-C subscales and total score between people with stroke with depression (GDS-15-C score ≥ 8) and without depression (GDS-15-C score < 8).

Subscale	Median (interquartile range)		Mann–Whitney U	Z	*P*
	Without depression (n = 40)	With depression (n = 17)			
Family	23.50 (12.00)	16.00 (14.00)	210.00	− 2.285	0.022*
Friends	19.00 (9.00)	15.00 (11.00)	236.50	− 1.810	0.070
Significant other	20.00 (9.00)	15.00 (12.00)	217.50	− 2.141	0.032*
Total score	59.00 (25.00)	45.00 (29.00)	215.50	− 2.173	0.030*

*p* < 0.05*.

**Table 7:** Comparisons of MSPSS-C subscales and total score between people with stroke with low (Berg Balance Scale score ≥ 45) and high fall risk (Berg Balance Scale score < 45).

Subscale	Median (interquartile range)		Mann–Whitney U	Z	*P*
	Low fall risk (n = 43)	High fall risk (n = 14)			
Family	23.00 (8.00)	11.50 (15.00)	141.00	− 2.988	0.003**
Friends	19.00 (9.00)	15.50 (15.00)	218.50	− 1.533	0.125
Significant other	20.00 (10.00)	16.00 (14.00)	209.00	− 1.709	0.087
Total score	58.00 (29.00)	42.00 (34.00)	168.50	− 2.458	0.014*

*p* < 0.05*, *p* < 0.01**.

## Discussion

To the best of our knowledge, this is the first study to investigate the psychometric properties of the MSPSS-C in people with stroke. In this study, the MSPSS-C showed excellent internal consistency, but a ceiling effect was detected in the family subscale. The instrument had a two-factor structure, with its total score demonstrating significant weak to moderate correlations with the Brief 2-Way SSS-C, BBS, GDS-15-C, and CIM-C scores. Compared with people without stroke, those with stroke experiencing moderate to very severe motor impairments had significantly lower scores on the total scale and the subscales of friends and significant other. In addition, among people with stroke, those who had depressive symptoms and high fall risks had lower MSPSS-C scores than those with no depressive symptoms and low fall risks.

The MSPSS-C subscales demonstrated excellent internal consistency in this study (Cronbach’s α = 0.94–0.95). The Cronbach’s α values obtained in our study are better than those previously obtained for the Hausa version of the MSPSS (Cronbach’s α = 0.78–0.90) in a study of Nigerian people with stroke^12^. This suggests that some items in the MSPSS-C operationalised the construct of receiving social support by using overlapping items. For example, both item 3 (‘My family really tries to help me’) and item 11 (‘My family is willing to help me make decisions’) assessed the perception of receiving help from family, but item 3 was a generic concept that could refer to any aspect and item 11 more specifically referred to decision making. This overlapping may have resulted in a higher degree of internal consistency in the MSPSS-C. The Cronbach’s α values obtained in this study were over 0.9, indicating that some items of the MSPSS-C were potentially redundant^42^. High Cronbach’s α values for the MSPSS have also been reported in studies involving people with comorbid chronic obstructive pulmonary disease and heart failure (Cronbach’s α = 0.92–0.95)^17^ and chronic diseases (Cronbach’s α = 0.91–0.96)^43^. Therefore, future studies are recommended to eliminate or modify redundant items of this scale.

The ceiling effect detected in the family subscale of the MSPSS-C may be explained by considering the characteristics of the sample included in this study. Most of the participants were married (77.19%) and lived with family members (87.72%); in Chinese families, people often receive high levels of support of various kinds from their family members. This finding of a ceiling effect is consistent with that reported for the Swedish and Korean versions of the MSPSS, for which ceiling effects were also reported in the family subscales due to participants receiving overall high support from family members^18,44^.

Among the people with stroke, the perceived social support measured by MSPSS-C exhibited a significant weak to moderate correlation with the Brief 2-way SSS-C. The possible explanation is that while MSPSS-C focuses on the aspect of perceived social support, the Brief 2-way SSS-C measures both the aspects of receiving and giving of social support. Thus, there was only weak to moderate correlation between the MSPSS-C and Brief 2-way SSS-C as the construct of social support was conceptualised differently in these 2 outcome measures. These findings also highlighted the discriminant validity that the Brief 2-way SSS-C and MSPSS-C measured different constructs.

As expected, our results demonstrated a significant positive correlation between perceived social support and community integration, in line with the results of a previous study on a stroke population^45^. This correlation can be explained by the fact that high levels of perceived social support can enhance the social involvement of people with stroke, which in turn facilitates better community integration^45^.

Of note, the mental health-related quality of life (MCS in the SF-12-C) of our participants was significantly correlated with their scores on the family subscale, but not their scores on the friends and significant other subscales, of the MSPSS-C. This correlation may be explained by the cultural and social constructs of Chinese society. Traditional Chinese culture emphasises close family relations, in which senior members of the family should be taken care of by siblings and younger family members^46^. This explains why the family has a greater impact on perceived social support than do friends or significant others in people with stroke. Similar findings have been reported for older adults^46^. In contrast, no significant correlation was found between the MSPSS-C scores and physical health-related quality of life (PCS in SF-12-C) of our participants. The physical health-related quality of life has been demonstrated to be associated only with emotional and financial support in people with stroke^47^, whereas the perception of social support can involve instrumental, informational, and appraisal support from others, in addition to emotional and financial support. This explains why the MSPSS-C scores were not correlated with the PCS scores in the SF-12-C.

Further, perceived social support was significantly correlated with the BBS scores in our study. Just as high levels of perceived social support enhance the functional status of people with stroke^7,11^, higher perceived social support can also increase compliance with long-term treatment or participation in rehabilitation programmes among people after stroke^48^, which can in turn facilitate their motor recovery and improve balance performance and fall risks. In contrast, the MSPSS-C scores showed nonsignificant correlations with the motor impairment measure (the FMA score) and the functional mobility measure (the TUG completion time). The insignificant association between MSPSS-C and FMA might be explained by the compensatory strategies adapted by community-dwelling stroke survivors. Despite the fact that the perceived social support could affect the functional performance of people with stroke^7,11^, adaption of various compensatory motor patterns can accommodate their motor impairment after stroke and support their functional recovery. Thus, insignificant correlations between perceived social support and motor impairment in this cohort of community-dwelling and cognitively intact people with chronic stroke might occur. On the other hand, TUG is a tool to examine the functional mobility of people with stroke through the tasks of sit-to-stand, walking and turning. Although the TUG consists of common motor tasks and simple movements, it also require executive ability of the subjects for optimal performance^49^. This reflects that TUG is not only evaluating the motor performance, but it is also assessing cognitive function of the individual. Due to the complex characteristics of TUG, even though the perceived social support was associated with the functional status of people with stroke^7,11^, discrepancies might also exist between the perception of social support and the results of TUG. In addition, motor function alone is insufficient in predicting the level of perceived social support of people with stroke. Other factors, including post-stroke depression and stroke recovery, may affect the level of perceived social support in stroke populations.

In this study, a two-factor structure for the MSPSS-C was determined, reflecting two relatively independent dimensions of social support—‘friends’ and ‘family’—among people with stroke. Our result was consistent with the two-factor structure of the MSPSS for Chinese adolescents determined by Chou et al.^22^, but not with the three-factor structure of the original version of the MSPSS. In fact, some other studies^2,12,22^ have also reported a two-factor structure for the MSPSS, with the ‘significant other’ subscale merged into the ‘family’ or ‘friends’ subscale. These inconsistencies between different studies may be due to linguistic and socio-contextual variances in cross-cultural translations of the MSPSS^44^. For example, the term ‘a special person’ in the original version of the MSPSS was not precisely defined; this term can be interpreted in different ways based on the cultural background^44^. In the MSPSS-C, the term ‘a special person’ was replaced by ‘a best friend’. Thus, the ‘significant other’ subscale in the original version was loaded on the ‘friends’ factor in the MSPSS-C.

In our study, people with stroke had significantly lower total MSPSS-C scores than those without stroke. The perception of social support is primarily associated with day-to-day issues that individuals experience. Sensorimotor impairments, functional limitations, impaired cognition, and emotional changes following stroke tend to reduce social integration and interpersonal interaction^50,51^. People after stroke often find difficulties in performing activities required for reintegration into community or social networks, such as socialising, going out, and travelling^52^, which could negatively affect their subjective perceptions of social support in daily life situations.

When the scores of each MSPSS-C subscale were compared between people with and without stroke in our study, significantly lower perceived social support was found in the subscales of ‘friends’ and ‘significant other’, but not ‘family’, in people with stroke. This may be due to a reduction of social participation or social networking due to physical limitations caused by stroke. A nonsignificant difference was found in the ‘family’ subscale scores between people with and without stroke. As the majority of our participants with stroke (87.7%) and without stroke (90%) lived with their family members, both groups could obtain essential support from their family and thus demonstrated comparable levels of perceived social support from family.

Among people with stroke, the levels of perceived social support from family and significant others were significantly lower in participants with depressive symptoms than in those without depressive symptoms. People after stroke might have less social contact with friends due to physical limitations, so their primary source of perceived social support would be family and their significant other. When people with stroke perceive low level of social support from family and their significant other, they may experience social isolation and feelings of loneliness^53^. Emotional loneliness can result in distress and apprehension and increase the risk of depression^54^.

Interestingly, among people with stroke, perceived social support from family was significantly lower in participants with higher fall risks than in those with lower fall risks. Family members are the main carers providing instrumental support to assist with transportation and social participation for stroke survivors^55^. Participation in social activities and rehabilitation programmes can improve the motor recovery and functional performance of people with stroke. Consistent with our finding, another study showed that high levels of perceived social support from family improved the functional status of people with moderate or severe stroke^11^. Functional performance directly affects the balance ability and fall risk in people with stroke. Thus, higher perceived social support in people with stroke might reduce their risk of fall.

This study has several limitations. First, the participants with stroke in this study were recruited from local self-help groups. They were cognitively intact and had good functional mobility to travel and participate in the study; accordingly, they may have had high levels of social support from their peers. Thus, the applicability of our findings may be limited to people with stroke who fulfil the inclusion and exclusion criteria used in this study. Second, the sample size was barely adequate for prinicipal component analysis in this study, and confirmatory factor analysis was not performed. Therefore, we recommend that future studies perform confirmatory factor analysis to further examine the two-factor structure of the MSPSS-C. Third, social networking and perceived social support might differ across genders^56^; thus, the uneven male–female ratio may have affected the results of this study. Fourth, we assessed only 50 people with stroke although the planned sample size was 51. It was because one of the people with stroke could not attend the assessment due to dizziness. However, we believed that missing 1 participant would have minimal influences as we had prudently estimated the required sample size. Finally, we did not examine the test-retest reliability and predictive validity of MSPSS-C. We recommend future studies to further investigate these aspects.

### Clinical implications

The MSPSS-C can be applied in clinical settings to measure perceived social support in stroke survivors. Healthcare providers may consider using it to evaluate the effectiveness of interventions aimed at rehabilitating stroke survivors. Attention should be paid to stroke survivors with depressive symptoms and high fall risks because these factors are correlated with reduced levels of perceived social support.

## Conclusion

The MSPSS-C is a valid tool for assessing perceived social support in community-dwelling chronic stroke survivors with moderate to very severe motor impairment. Our study suggests that the levels of perceived social support are significantly lower in community-dwelling people with stroke than in those without stroke. To improve the functional recovery, community integration, and mental well-being of people with stroke, clinicians should consider implementing strategies to enhance the levels of perceived social support in this population.

## Data Availability

The data that support the findings of this study are available from the corresponding author upon reasonable request.
